# Research and Development of Parameter Extraction Approaches for Memristor Models

**DOI:** 10.3390/mi12101220

**Published:** 2021-10-06

**Authors:** Dmitry Alexeevich Zhevnenko, Fedor Pavlovich Meshchaninov, Vladislav Sergeevich Kozhevnikov, Evgeniy Sergeevich Shamin, Oleg Alexandrovich Telminov, Evgeniy Sergeevich Gornev

**Affiliations:** 1 Moscow Institute of Physics and Technology, 9 Institutskiy per., Dolgoprudny, 141701 Moscow, Russia; DmitryZhev@yandex.ru (D.A.Z.); kozhevnikov.vs@phystech.edu (V.S.K.); yevgeniy.shamin@phystech.edu (E.S.S.); otelminov@niime.ru (O.A.T.); egornev@niime.ru (E.S.G.); 2 Joint-Stock Company “Molecular Electronics Research Institute” (JSC MERI), 12/1 1st Zapadnyi Proezd, Zelenograd, 124460 Moscow, Russia

**Keywords:** memristor, compact modeling, volt–ampere characteristic, parameter extraction, optimization, machine learning

## Abstract

Memristors are among the most promising devices for building neural processors and non-volatile memory. One circuit design stage involves modeling, which includes the option of memristor models. The most common approach is the use of compact models, the accuracy of which is often determined by the accuracy of their parameter extraction from experiment results. In this paper, a review of existing extraction methods was performed and new parameter extraction algorithms for an adaptive compact model were proposed. The effectiveness of the developed methods was confirmed for the volt-ampere characteristic of a memristor with a vertical structure: TiN/Hf_x_Al_1−x_O_y_/HfO_2_/TiN.

## 1. Introduction

Research in the fields of energy-efficient memory and the development of neuromorphic systems in recent years has largely been related to research on memristors. A memristor [1,2] is a nonlinear element of an electric circuit in which resistance can reversibly change depending on the electrical signal entered as its input. Elements developed in recent years demonstrate the possibility of building complex high-performance devices, applicable to the construction of complex neural networks and multi-level logical elements, as well as to serve as ideal memory storage for high-speed real-time operations [3,4,5,6,7].

Depending on the type of memristive element used, the final structure inherits its main flaws, mostly related to the random nature of the processes occurring inside the device [8,9,10,11].

The random nature of processes limits accurate quantitative modeling of system evolution phenomena, and at the moment, a significant portion of existing models rely on constants derived from the analysis of experimentally obtained results. Thus, the model accuracy depends on the method and quality of parameter extraction.

Although the choice of specific techniques for parameter extraction from an experiment directly depends on the type and structure of the model, most of the existing methods can be divided into four main groups:

The first group consists of methods related to the analysis of individual parts of the volt-ampere characteristic for separate and sequential estimation of model parameters. These methods are mainly used in the following cases: the possibility of the direct solution of model equations [12], direct dependence of the model properties on the material characteristics measured during the experiment, and for constructing initial approximations to more complex methods [11,13].

The second includes various schemes of brute-force model parameter brute-force search over grids based on different assumptions. This approach is more or less applicable in all areas, from the construction of initial approximations to the selection of model hyperparameters.

The third group comprises of methods for constructing an optimization problem by approximating the key characteristics of the device response or its volt-ampere characteristic. The main differences in the approaches of this group are related to the choice of a characteristic for approximation, the construction of the error functional, and methods of data preprocessing and approximation [14,15,16].

The fourth includes machine learning methods for time series analysis, demonstrating promising results for the task of memristor model parameter extraction. Supervised learning is used as the main approach in tasks of this type, the complexity of which is mainly determined by the quality of the training dataset, which is constructed on the basis of the memristor model and datasets of previously obtained characteristics.

To the best of our knowledge, consideration of model selection is based primarily on the purpose for which the model is selected. The most promising in terms of modeling the dynamic properties of the memristor are physical models—for instance, models using the Kinetic Monte-Carlo method [8]. For practical applications, including schematic modeling of devices using a memristor, compact models (e.g., [11]) are the most common.

In this paper, the main groups of methods of memristor model parameter extraction are highlighted. For each group, examples of current approaches are given, their advantages and disadvantages are highlighted, and their areas of use are identified. Within the framework of a previously developed mobility modification model [11], new methods were developed, the joint application of which to the extraction of experimental parameters has improved the quality and speed of extraction for volt-ampere characteristic analysis. During the development and application of machine learning methods to the task of memristor parameter extraction, approaches to construct samples based on the existing memristor model were first described. A random forest algorithm built on the training dataset allowed for a fairly accurate estimation of model parameters, the use of which, as an initial approximation, allowed the acceleration of the solution of the optimization problem. All of the methods presented in this paper can be generalized to a subclass of adaptive compact models for the acceleration and improvement of the accuracy of parameter extraction results.

## 2. Materials and Methods

### 2.1. Experiment Description

The developed extraction methods were applied to the volt–ampere characteristic from a series of experiments first presented in [17]; the developed thin-film structure TiN/Hf_x_Al_1−x_Oy(6 nm)/HfO_2_(4 nm)/TiN was investigated.

**Figure 1:**
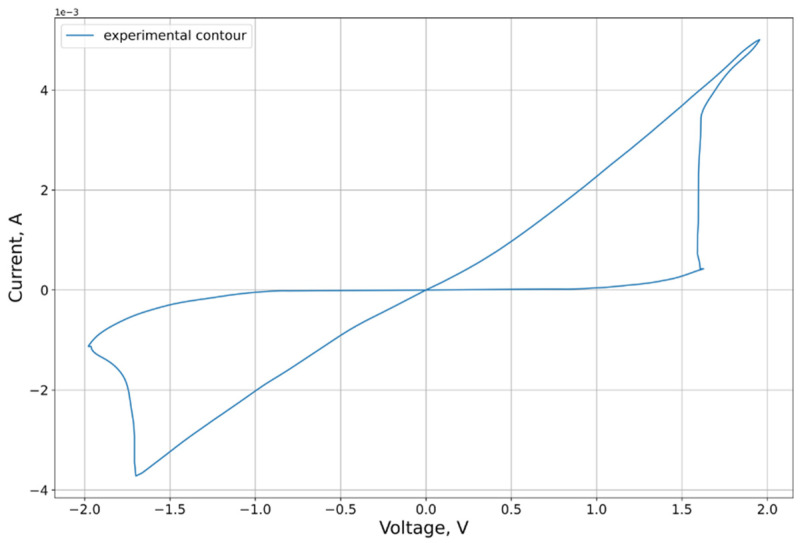
Analyzed sample of the obtained VAC of the memristor structure TiN/Hf_x_Al_1−x_Oy(6 nm)/HfO_2_(4 nm)/TiN.

An example of the characteristic obtained in the experiment is shown in Figure 1. An Agilent B1500A (Agilent Technologies, Santa Clara, CA, USA) characterizer including an Agilent B1530A (Agilent Technologies, Santa Clara, CA, USA) high-speed source meter, an Agilent B1517A (Agilent Technologies, Santa Clara, CA, USA) quasi-static volt-ampere characterization device, and an Agilent B1525A pulse generator were used as the main measurement equipment for constructing quasi-static volt-ampere characteristics.

An input voltage signal was applied to the upper TiN electrode, while the lower electrode was grounded. Sinusoidal signal with an amplitude of 2 V and frequency of 1000 Hz was applied during cycling.

On the one hand, the construction of a complex thin-film structure with the inclusion of hafnium dioxide allows one to obtain promising characteristics. On the other hand, the complex dynamics of physical and chemical processes within the multilayer structure limit the use of most of the currently developed physical and chemical memristor models—that is why the key approach to simulating the volt-ampere characteristics of such structures is compact modeling.

An adaptive model of mobility modification [11] was used to simulate the volt-ampere characteristic.

### 2.2. Model Description

As part of this work, we have performed the construction of various methods for extracting the parameters of the compact model of mobility modification proposed in [11], based on [18]. The compact adaptive model proposed for consideration by the authors involves simulation modeling of one volt-ampere characteristic. In the model, the existence of at least two distinguishable states (Ron, Roff) is provided by the equation structure.

The current–voltage relation and evolution equations in this model take the following form:

(1) $$i=\left(\Pi_{i=1}^{n}U_{i}\left(x\right)\right)\{\begin{array}{cc} a_{1}x\sinh\left(bv\right), & v>0\\ a_{2}x\sinh\left(bv\right), & v<0 \end{array},$$

(2) $$\frac{dx}{dt}=g\left(v\right)f\left(x,v\right)$$

(3) $$g\left(v\right)=\{\begin{array}{cc} A_{p}\left(e^{v}-e^{V_{p}}\right), & v>V\\ -A_{n}\left(e^{-v}-e^{V_{n}}\right), & v<-V_{n}\\ 0, & -V_{n}\leq v\leq V_{p} \end{array},$$

(4) $$f(x,\;v>0)=\{\begin{array}{cc} e^{-\alpha_{p}\left(x-x_{p}\right)}w_{p}\left(x,x_{p}\right), & x\geq x_{p}\\ 1, & x<x_{p} \end{array},$$

(5) $$f\left(x,\;v\leq 0\right)=\{\begin{array}{cc} e^{-\alpha_{n}\left(x+x_{n}-1\right)}w_{n}\left(x,x_{n}\right), & x\leq 1-x_{n}\\ 1, & x>x_{n} \end{array},$$

(6) $$w_{p}\left(x,x_{p}\right)=\frac{x_{p}-x}{1-x_{p}}+1,$$

(7) $$w_{n}\left(x,\;x_{n}\right)=\frac{x}{1-x_{n}}.$$

Here, x is the state variable, $a_{1,2}$ and b are constants, $A_{p}$ and $A_{n}$ are constants that determine the change rate of the state variable after overcoming threshold voltages, and $V_{p}$ and $V_{n}$ are the absolute values of the upper and lower threshold voltages, respectively. Parameters $x_{p}$ and $x_{n}$ are restricted only to the range [0, 1]. Parameter n is a hyperparameter representing the number of inhomogeneities taken into account, and function $U_{i}\left(x\right)$ is the accounting function of the inhomogeneities:

(8) $$U_{i}\left(x\right)=\{\begin{array}{cc} exp\left(-\frac{{\left(x-x_{i}\right)}^{2}}{2\sigma_{i}^{2}}\right), & x<x_{i}\\ 1, & x\geq x_{i} \end{array}.$$

In (8) $x_{i}$ is the effective position of the *i*-th inhomogeneity in the state space of a memristor, and $\sigma_{i}$ is the effective width of a given inhomogeneity.

### 2.3. Analytical Approaches and VAC Analysis by Parts

The first group of methods includes techniques for analyzing the results of experimentally obtained characteristics of memristive devices or structures, specifically designed to analyze the properties of the memristor functional layer. In general, all approaches differ significantly depending on the model and experimental structure used.

An example of a first-group method was considered in [19], where an approximation of a “flux + charge”-type model was conducted. The behavior of the memristor is described in this model in terms of flux and charge with the following formula:

(9) $$Q=Q_{0}\cdot {\left(\frac{\phi }{\phi_{0}}\right)}^{1+n},$$

where Q and ϕ are charge and flux respectively, $Q_{0}$ and $\phi_{0}$ are internal parameters, and n is a constant technological parameter, defined as:

(10) $$n=m\cdot \ln\left(1+\frac{\Delta G}{G_{m}}\right),$$

In the Equation (10), m is the pulse number, $G_{m}$ is the measured conductivity at step m, and ΔG is a conductivity change with respect to the previous pulse.

Models defined in “charge–flux” terms are also used to describe both individual memristors and circuits containing memristors [20].

A similar approach was proposed by Messaris et al. [12]; the basis of the method proposed by the authors is the analytical integrability of the evolution equation when signals of a rectangular form are applied.

The model equations have the following form:

(11) $$\frac{dR}{dt}=m\left(R,v\right)=s\left(v\right)f\left(R,\;r\left(v\right)\right),$$

(12) $$s\left(v\right)=\{\begin{array}{cc} A_{p}\left(-1+e^{\frac{\left|v\right|}{t_{p}}}\right), & v>0\\ A_{n}\left(-1+e^{\frac{\left|v\right|}{t_{n}}}\right) & v\leq 0 \end{array},$$

(13) $$f\left(R,\;v\right)=\{\begin{array}{cc} {\left(r_{p}\left(v\right)-R\right)}^{2}, & v>0\\ {\left(R-r_{n}\left(v\right)\right)}^{2}, & v\leq 0 \end{array},$$

(14) $$r\left(v\right)=\{\begin{array}{cc} r_{p}\left(v\right)=a_{0,p}+a_{1,p}v\;, & v>0\\ r_{n}\left(v\right)=a_{0,n}+a_{1,n}v, & v\leq 0 \end{array}.$$

When a constant voltage $V_{b}$ is applied, the dependence of resistance on time is described by the formula:

(15) $$R\left(t\right)=\frac{R_{0}+s\left(V_{b}\right)r\left(V_{b}\right)\left(r\left(V_{b}\right)-R_{0}\right)t}{1+s\left(V_{b}\right)\left(r\left(V_{b}\right)-R_{0}\right)t},$$

where $R_{0}$ is the initial resistance.

The main idea of this method includes the separated estimation of model parameters based on applied voltages. A sequence of rectangular pulses of different signs with gradually increasing amplitudes is applied to the input of the memristor. The first switching cycle with a pronounced trend is used to extract the parameters of function s(v) and the value of function $r\left(V_{b}\right)$, while the remaining cycles allow the values of function r(V) parameters to be obtained using the previously defined parameters. Thus, by applying a sequence of pulses with gradually increasing amplitudes, it is possible to estimate the values of the model parameters. A similar approach is also presented in [21].

In addition to performing specific experiments, volt-ampere characteristic analysis is also used to estimate the parameters of the memristor model. For example, the first derivative method was applied by Yakopcic et al. when analyzing the VACs obtained on a TaOx structure [13]. In this approach, the threshold voltage was defined as the global extremum of the current–voltage derivative. This paper includes the extraction of the parameters of the evolution equation based on $g_{min}$ and $g_{max}$ as defined in:

(16) $$i\left(t\right)=g_{max}v\left(t\right)x\left(t\right)+g_{min}\sinh\left(b\;v\left(t\right)\right)\left(1-x\left(t\right)\right).$$

Here, the parameters $g_{min}$, $g_{max}$ and b are extracted from the volt-ampere characteristc using the least-squares regression algorithm. It is assumed that the state variable x is equal to one in the “on” state and zero in the “off” state. Considering the differential conductivity dependence on time, the authors determine three features: the maximum and minimum rates of conductivity change, and conductivity values at a sharp deceleration of conductivity change; the resulting features are proposed for use in the estimation of the parameters $A_{p}$, $A_{n}$, $x_{p}$, $x_{n}$. As noted by the authors, the algorithm was developed for VACs with clear transitions between regions with the presence and absence of state evolution. It is important to note that this algorithm may not work well on noisy contours and requires data preprocessing.

### 2.4. Brute Force Methods

In general terms, the problem with grid searches of parameters is that they are a computationally expensive way to solve the discrete optimization problem. In spite of this, this method has become widespread for solving the problem of memristor parameter extraction. One of the applications of this approach is the estimation of model hyperparameters. The use of hyperparameters in memristor models can solve a wide variety of subtasks; for example, in a physical model, they can be related to the choice of the shape of the conducting region [22]—or for a compact model using a window function, to the choice of the optimal window function [23]. Another possibility is cases where a number of model parameters take a discrete set of values; for example, in physical models, the problem may involve selecting tabular values for the material [9,24,25].

In addition, this method is used in optimization problems where the combination of the model and the error functional limits the use of other approaches. In these problems, the brute-force search usually aims to estimate a part of the parameters [14] in order to simplify further optimization. In addition to the estimation of a part of the parameters, brute-force search over a sparse grid is used to select several initial approximations, which is useful for compensating for errors related to using local optimization methods.

### 2.5. Local Optimization Methods

This method represents the most common approach for the approximation of volt-ampere characteristics, as it does not require additional experiments, is less sensitive to variations in characteristics than the first-group methods, works orders of magnitude faster than brute-force search, and, unlike machine learning, does not require either construction of a training dataset or a pretrained model. The accuracy of its VAC approximation depends on the target function and the chosen combination of optimization methods.

#### 2.5.1. Building the Target Function

**Figure 2:**
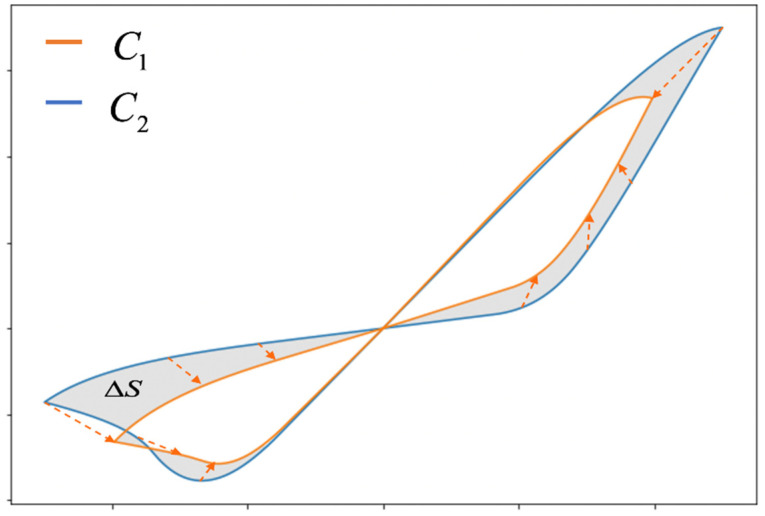
Schematic representation of the absolute symmetric difference of the area of figures $S\left(C_{1}\Delta C_{2}\right)$ limited by contours $C_{1}$ and $C_{2}$, shown as the area of the shaded region.

To describe the differences between the real curve and the model curve, the MSE between selected points of the volt-ampere characteristic is usually used. Additionally, it is possible to use an estimate of the difference in power characteristics of the VACs based on measurement of the normalized symmetric difference area of the VACs (Figure 2):

$$\Delta S=\frac{S\left(C_{1}\Delta C_{2}\right)}{S\left(C_{1}\right)},$$

where $C_{1}$ and $C_{2}$ are approximating and approximated contours respectively, $C_{1}\Delta C_{2}$ is the symmetric difference of the regions bound by contours $C_{1}$ and $C_{2}$, and $S\left(C_{2}\right)$ is the area of the region bound by experimental contour $C_{2}$.

#### 2.5.2. Approaches to Volt-Ampere Characteristic Approximation

The approximation of a single, experimentally derived volt-ampere characteristic is usually not very difficult and is usually done using computationally expensive full brute-force methods in combination with simple gradient methods; for instance, in [14], where a combination of brute-force was used together with the stochastic gradient descent method. Another example of local and global approximation methods usage is presented in [16].

In addition to approximation methods, “log-antilog”, scaling, smoothing, and state equation preprocessing [15] methods can also be used to improve the accuracy of the final approximation and numerical integration of the evolution equation.

### 2.6. Machine Learning Approaches

One of the most promising areas of research in model parameter extraction is the use of machine learning. The approaches in [26,27,28,29,30,31,32,33] which are related to its use include both improving the efficiency of other extraction methods through speeding up the calculation of differential equations or prediction of the volt-ampere characteristic, and direct parameter extraction from an experimental VAC.

At the moment, there is active development of machine learning application to the analysis of well-studied devices, but for the memristor, due to its special properties, this direction is only beginning to develop [34,35], and is used to predict memristor behavior during cycling.

Attempts to use machine learning for parameter extraction are limited by a number of serious problems related to the fundamental properties of the structures under consideration. The type of memristor and the structure of the applied model affect all essential subtasks: the construction of datasets, the definition of the target function, and the choice of machine learning model structure.

In general, there is no possibility of conducting a sufficient number of different experiments to form a dataset. As such, we propose to use a model pre-trained on a synthetic dataset. Thus, the construction of a machine learning model for parameter extraction is a supervised learning task, which includes the need to define a set of rules for constructing a training dataset.

By solving the inverse problem for a certain model, the sample can be constructed on its basis using a signal corresponding to the experimental one. Its construction includes two main tasks: estimation of parameter interrelations to obtain physically significant results and estimation of acceptable ranges of variation of model parameters. It is possible to select two approaches to these problems:

The first is based on determining the relationships between model parameters and curve characteristics in order to select specific parameter sets corresponding to plausible I–V curves. This approach, in addition to the limitations associated with analytical or computational solution, has a number of drawbacks. First of all, there is the lack of verified criteria for the plausibility of volt-ampere characteristics built on the basis of the internal dependences of the model parameters for an arbitrary structure.

The second approach is more resource-intensive, because it involves generating random volt-ampere characteristics and testing for their correspondence to the characteristics of a particular class of devices. In this paper, the second approach was used.

## 3. Results

New analytical methods for parameter estimation were developed for the model presented for analysis. The demonstration of the developed algorithms is carried out for the volt-ampere characteristic presented in Section 2.1 (Figure 1).

### 3.1. Series Expansion

In the case of the considered model, the series expansion made it possible to estimate the parameters $a_{1,2}$ and b. Considering the low resistance branch of the right lobe of the VAC, this region can be cut off—in which case, g(v) is known to be non-zero. In the remaining area 100 points of the low resistance branch with uniform voltage step ${\left\{\left(v_{k},\;i_{k}\right)\right\}}_{k=0}^{100}$ are taken; then, the parameter search problem is reduced to minimization of MSE. Assuming that in the low-resistance state, x is equal to one, we obtain two equations allowing us to calculate the required parameters:

(17) $$\underset{k}{\sum }v_{k}i_{k}\cosh bv_{k}-\frac{\sum_{k}i_{k}\sinh bv_{k}}{\sum_{k}\sinh^{2}bv_{k}}\underset{k}{\sum }\sinh2bv_{k}=0,$$

(18) $$a_{1}=\frac{\sum_{k}i_{k}\sinh bv_{k}}{\sum_{k}\sinh^{2}bv_{k}}.$$

Here, the criterion of extremum of the function $MSE\left(a_{1},\;b\right)$ was deliberately not specified, since in practice it often turns out that outside the switching region one can distinguish a sufficiently large region in which the condition bv≪1 is satisfied. The upper bounds of these regions can be determined using the algorithms for threshold voltage estimation.

Expanding Equations (18) and (19) up to the fifth order of bv, the following is obtained:

(19) $$b^{4}A+b^{2}B+C=0$$

(20) $$A=\frac{1}{24}\underset{k}{\sum }i_{k}v_{k}^{4}+\;\frac{\sum_{k}v_{k}\cdot \left[\frac{1}{120}\sum_{k}i_{k}v_{k}^{5}-\frac{1}{18}\frac{\sum_{k}i_{k}v_{k}^{3}\sum_{k}v_{k}^{4}}{\sum_{k}v_{k}^{2}}+\frac{1}{9}{\left(\frac{\sum_{k}v_{k}^{4}}{\sum_{k}v_{k}^{2}}\right)}^{2}\sum_{k}i_{k}v_{k}\right]+\frac{2}{15}\sum_{k}v_{k}^{5}\sum_{k}i_{k}v_{k}}{\sum_{k}v_{k}^{2}}$$

(21) $$B=\frac{1}{2}\underset{k}{\sum }i_{k}v_{k}^{2}+\frac{\frac{1}{6}\left(\sum_{k}i_{k}v_{k}^{3}-2\frac{\sum_{k}v_{k}^{4}}{\sum_{k}v_{k}^{2}}\sum_{k}i_{k}v_{k}\right)\sum_{k}v_{k}+\frac{2}{3}\sum_{k}v_{k}^{3}\sum_{k}i_{k}v_{k}}{\sum_{k}v_{k}^{2}}$$

(22) $$C=\underset{k}{\sum }i_{k}+\frac{\sum_{k}i_{k}v_{k}\sum_{k}v_{k}}{\sum_{k}v_{k}^{2}}$$

(23) $$a_{1}=\frac{1}{b\sum_{k}v_{k}^{2}}\left[\underset{k}{\sum }i_{k}v_{k}+\frac{1}{6}b^{2}\left(\underset{k}{\sum }i_{k}v_{k}^{3}-2\frac{\sum_{k}v_{k}^{4}}{\sum_{k}v_{k}^{2}}\underset{k}{\sum }i_{k}v_{k}\right)\;+b^{4}\left(\frac{1}{120}\underset{k}{\sum }i_{k}v_{k}^{5}-\frac{1}{18}\frac{\sum_{k}i_{k}v_{k}^{3}\sum_{k}v_{k}^{4}}{\sum_{k}v_{k}^{2}}\;+\frac{1}{9}{\left(\frac{\sum_{k}v_{k}^{4}}{\sum_{k}v_{k}^{2}}\right)}^{2}\underset{k}{\sum }i_{k}v_{k}\right)\right]$$

Thus, using this decomposition, an estimate of the parameters b and $a_{1}$ can be obtained. Despite the cumbersomeness of the obtained formulas, the algorithm behind them is a simple computational task.

The resulting decompositions give the following estimates: $a_{1}=3.96\times 10^{-3}\;A$, $a_{2}=3.56\times 10^{-3}\;A$, and $b=0.54\;V^{-1}$.

**Figure 3:**
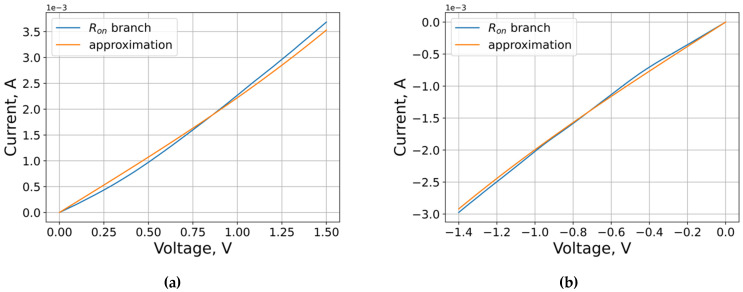
The results of the low-resistance branch approximation: (**a**) positive voltages; (**b**) negative voltages.

The results of the low-resistance branch approximation are shown in Figure 3.

However, if the accuracy of the preliminary estimate is important, it is better to use a nonlinear least-squares regression algorithm.

### 3.2. Assessment of Threshold Voltages

**Figure 4:**
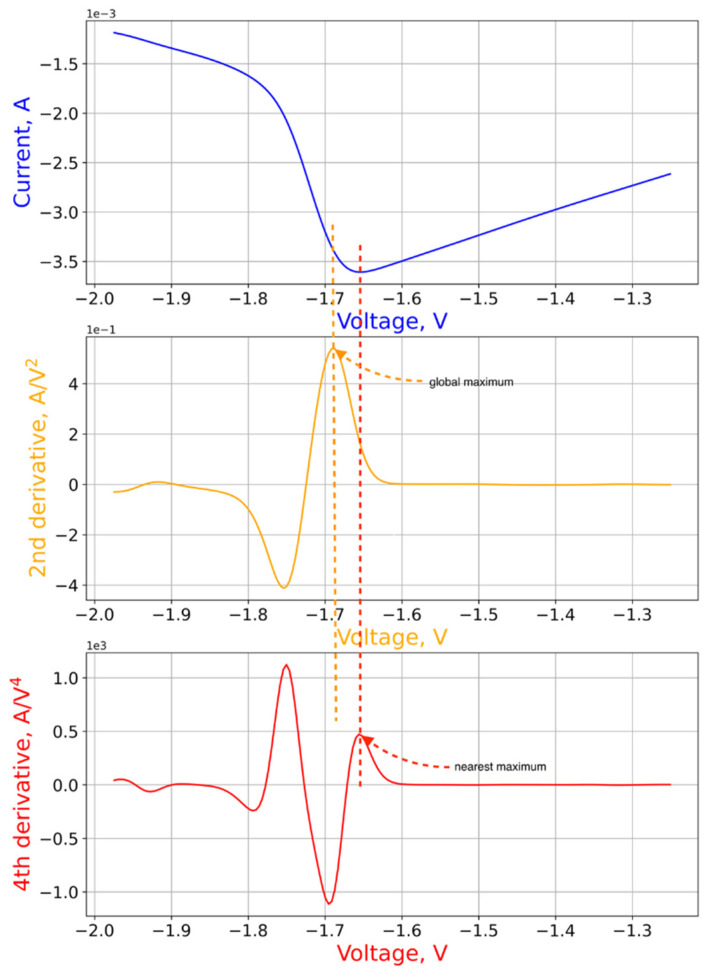
Application of the second derivative criterion to find the negative threshold voltage.

The position of the threshold voltage can be estimated using the second and fourth derivatives. The effect of this criterion is demonstrated visually in Figure 4. To find the threshold voltage, according to this criterion, it is necessary to determine the direction of the traversal beforehand. The forward direction of the circuit traversal results in an order of points at which the resistance decreases in the “positive” region of the VAC, and increases in the negative region. The opposite direction of the traversal corresponds to the opposite situation. In this paper, we considered the forward traversal direction (Figure 1).

The second step of the algorithm is the partition of the VAC into low-resistance and high-resistance branches with subsequent interpolation by polynomials or kernel smoothing. The endpoints of each branch are extreme right and left points of the VAC, and the choice in favor of one or another method of approximation is determined by the number of points in the experimental loop.

The third step is the calculation of the second and fourth derivatives of the current with respect to the voltage using the finite difference method. To determine the positive threshold voltage, it is necessary to consider the high resistance branch, determine the position of the global maximum of the second derivative and the nearest-to-maximum of the fourth derivative, which is to the left of the maximum of the second derivative. This step is empirically justified, since on average it gives a deviation from the optimum value of no more than 0.1 V. The negative threshold voltage is estimated in a similar way, with the high resistance branch replaced by a low resistance branch.

**Figure 5:**
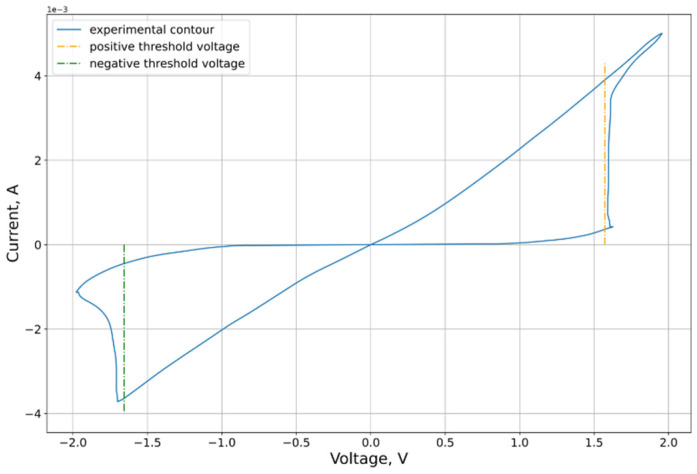
Approximated contour with marked threshold voltages.

On the VAC curve under consideration, the threshold voltage estimation algorithm gives the following parameter estimates: $V_{p}=1.57$ V, $V_{n}=1.66\;V$. The graph of the VAC with marked threshold voltages is shown in Figure 5.

### 3.3. Hyperparameter Estimation

**Figure 6:**
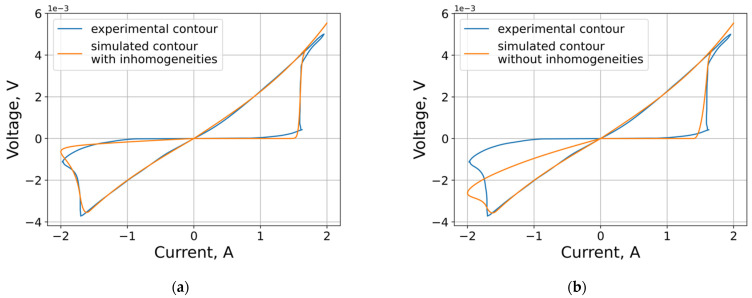
Model VACs with different numbers of inhomogeneities: (**a**) four inhomogeneities; (**b**) no inhomogeneities.

In the described model, the number of inhomogeneities can be considered as hyperparameters. The contribution of inhomogeneities to the shape of the VAC is demonstrated in Figure 6. The optimal number of inhomogeneities is four.

### 3.4. VAC Approximation and Dynamic Attractor Calculation

We used a combination of brute-force, multi-start [36], random search [37,38,39], BFGS [40], and Nelder–Mead’s methods [41] to obtain the best approximation result. The normalized difference area metric was chosen as the target function.

**Figure 7:**
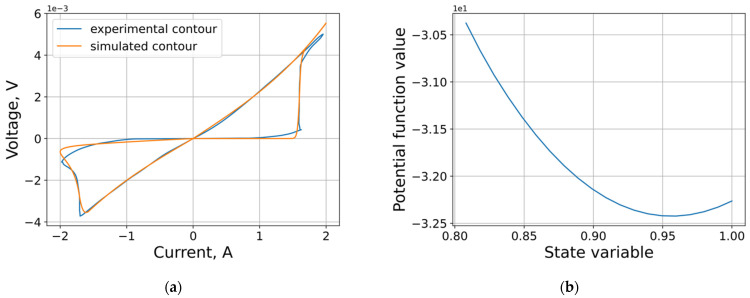
Approximation results: (**a**) model VAC [17]; (**b**) corresponding potential function.

The results of approximation and corresponding parameters obtained in the extraction are shown in Figure 7 and Table 1. The calculated model parameters were used to construct [42] the potential function of the memristor and calculate its dynamic attractor.

### 3.5. Machine Learning Approach

**Figure 8:**
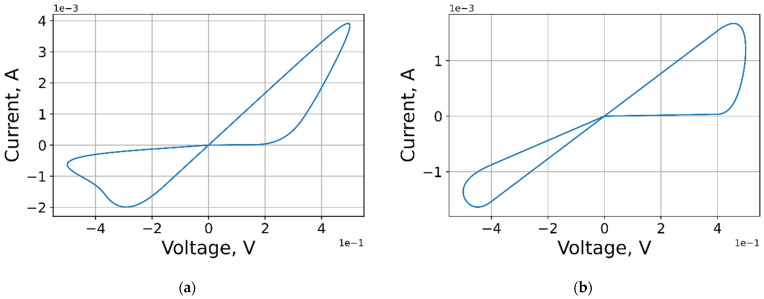
Examples of VACs from the dataset with parameters in: (**a**) row in Table 3; (**b**) row in Table 3**.**

It is worth noting that the choice of ranges is unique for each model and depends on the physical parameters of the materials used, environmental conditions, control signal, etc. However, in this work, we have defined the widest possible sampling ranges for the parameter sets (Table 2 and Table 3, Figure 8) in order to build the most general parameter extraction model, the accuracy of which can be improved. As class constraints for such a model, we used the following criteria for the adequacy of I–V curves: lack of artifacts (self-intersections, zero area of the VAC lobe, etc.) and experimentally determined forms of entering signal.

### 3.6. Model Training and Application

In our work, we used the random forest model of the Scikit-learn library [43]. This choice is due, on the one hand, to the reduction of possible overtraining, and on the other hand, to the possibility of parallel training.

The values of current and differential conductivity for ten points of each branch of the volt-ampere characteristic served as features of the object. The locations of the points are proportional to the maximum voltage for each branch and are located at the points of the curve corresponding to the interval $\left[0.1\;V_{max},1\;V_{max}\right]$.

The error function for supervised machine learning is constructed in the model parameter space and is based on minimizing the empirical risk between the predicted parameter vector and the real one constructed during sampling. In this paper, we used MSE as a more outlier-sensitive metric to increase the contribution of rare volt-ampere features in the model:

(24) $$Q\left(\alpha ,\;X^{l}\right)=\overset{l}{\underset{i=1}{\sum }}{\left(f\left(x_{i},\;\alpha \right)-y_{i}\right)}^{2}\;\to \;\underset{\;\alpha }{\min}\;,$$

where $\alpha \;\epsilon {\mathbb{R}}^{p}$ is the model parameter vector, $x_{i}$ is the feature vector of object i, $f\left(x_{i},\;\alpha \right)$ is the nonlinear regression model, and $y_{i}$ is the target value of object i.

Using the sampling method described earlier, a dataset size of 400,000 VAC contours was constructed; training and test datasets were 90% and 10% of the dataset, respectively. The size of this sample was sufficient to achieve the error functional minimum on the dataset of this configuration, due to the high internal dependencies of the contour features and the groups of described model parameters. Determination of hyperparameters and estimation of model accuracy was performed at cross validation with three folds.

**Figure 9:**
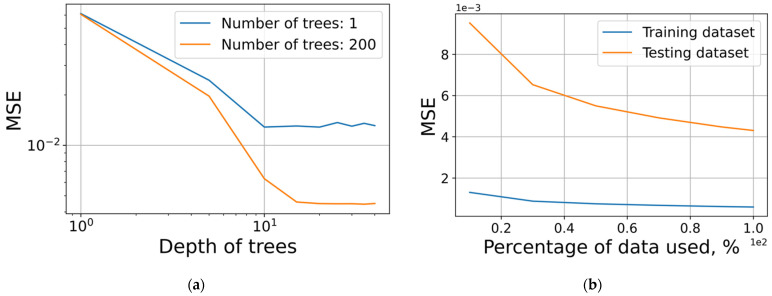
MSE loss function for: (**a**) different number and depth of trees; (**b**) percentage of dataset used in training and test.

The best depth of the forest and the number of its trees were 26 and 160; other random forest parameters remained at default. The resulting MSE loss function values for different forest characteristics and percentage of dataset used in training and test are presented in Figure 9.

As a result, the trained model was applied to the extraction of the parameter group from the experimental curve, and the results of the predictions are presented in Table 4. Although some of the predicted parameters are significantly different from the optimal ones (Table 4), using them as initial values for other approximation methods for VAC allowed us to abandon the multi-start method, obtaining comparable results.

This approach to training sample construction may seem redundant for a single VAC analysis. However, the application of this approach to multiple memristor switchings allows reduction of the optimization time, avoiding the use of global methods and related heuristics.

The influence of memristor device performance in the proposed model is taken into account in the construction of the training dataset. In the proposed approach, the training dataset is generated based on a compact adaptive model. Due to the rules of feature generation and the possibility of close samples in the parameter space, we can say that the system adequately responds to cycle-to-cycle changes and takes non-linear features into account.

The approximation time in the developed machine learning model is more than four times faster than the conventional approximation approach (Section 3.4): 136.6 s versus 621 s on our processor (Intel^®^ Core™ i7-9700K, TSMC, Hsinchu, Taiwan). Optimal parameters obtained with the machine learning model and optimization algorithms are presented in Table 5. The difference between these parameters and those in Table 1 are insignificant. The methods used can be applied in the analysis of a wide range of characteristic memristor CVCs [44,45,46,47].

## 4. Discussion

The classification of parameter extraction methods is presented in this paper. Methods related to analysis of model equations and individual parts of experimentally obtained characteristics, including various switching series. The techniques of this group allow one to make separate and sequential estimation of model parameters. The second group methods use schemes of brute-force search over parameter grids. The third group comprizes local optimization methods utilized to approximate the volt-ampere characteristics of the device. The fourth includes machine learning algorithm application to I–V curves, demonstrating promising results for the task of memristor model parameter extraction. For each group, examples of extraction algorithms are proposed, and their advantages and disadvantages are highlighted.

Within the framework of the previously developed mobility modification model [11], new methods were developed, the joint application of which to the extraction of experimental parameters has improved the quality of approximation. Decomposition of model equations in a series, selection of key areas of the VAC, and analysis of its shape allowed an algorithm for estimating threshold voltages and parameters of the current–voltage relation to be derived. For the optimization problem, it is suggested that the sequence of local methods using the multi-start method be considered.

To the best of our knowledge, there are no widely known papers with machine learning models that focus on memristor model parameter extraction. We propose a machine learning model to determine the initial approximation. The construction of the machine learning model included a description of the main approaches to the construction of the dataset. As a part of this work, the dataset was built within the framework of a described adaptive model with random parameters from specified intervals. Despite the general form of the training sample, the results of the random forest built on its basis allow one to obtain a good estimate of the initial approximation for other methods and demonstrate the promise of this approach for solving the extraction problem.

The methods of extraction of compact model parameters presented in this review and developed by the current authors can be applied to the analysis of a wide range of structures with a characteristic looped CVC. The development of unique methods for model parameter extraction for experiments aimed at studying a particular feature of the structure is a promising area in which the authors will continue their work.

It is important to note that regardless of the model chosen, considered approaches have a general nature and can mostly be applied to parameter extraction from arbitrary experiments (in particular, approaches in terms of optimization and machine learning based approaches).

## Figures and Tables

**Table 1:** Optimal parameters of the analyzed model with normalized symmetric difference area as error function.

Parameter	Value	Parameter	Value	Parameter	Value	Error Function
** $V_{p},\;V$ **	1.40	$a_{1},\;A$	$3.14\times 10^{-3}$	$x_{0}$	0.10	0.096
** $V_{n},\;V$ **	1.57	$a_{2},\;A$	$2.79\times 10^{-3}$	$x_{1}$	0.25	
** $A_{p}$ **	7357	$b,\;V^{-1}$	0.68	$x_{2}$	1.0	
** $A_{n}$ **	2068	$x_{start}$	$1.00\times 10^{-3}$	$x_{3}$	0.16	
** $x_{p}$ **	0.80	$\sigma_{0}$	0.25			
** $\;x_{n}$ **	0.17	$\sigma_{1}$	0.61			
** $\alpha_{p}$ **	0.71	$\sigma_{2}\;$	0.29			
** $\alpha_{n}$ **	11.19	$\sigma_{3}$	1.56			

**Table 2:** Chosen ranges of the model parameters in training set.

Parameter	Lower Bound	Upper Bound
** $a_{1},\;a_{2},\;A$ **	0	5
** $b,\;V^{-1}$ **	0	1
** $A_{p},\;A_{n}$ **	10	5000
** $V_{p},\;V_{n},\;V$ **	0	3
** $\alpha_{p},\;\alpha_{n}$ **	0	20
** $x_{0},x_{p},\;x_{n},\;x_{start}$ **	0	1
** $\sigma_{0}$ **	0	1

**Table 3:** Sets of parameters for Figure 8.

**Curve**	** $V_{p},\;V$ **	** $V_{n},\;V$ **	** $A_{p}$ **	** $A_{n}$ **	** $x_{p}$ **	** $x_{n}$ **	** $\alpha_{p}$ **	** $\alpha_{n}$ **	** $a_{1},\;A$ **	** $a_{2},\;A$ **	** $b,\;V^{-1}$ **	** $x_{start}$ **	** $x_{0}$ **	** $\sigma_{0}$ **
(**a**)	0.16	0.15	4000	4000	0.3	0.5	1	5	0.17	0.17	0.05	0.01	0	1
(**b**)	0.4	0.4	3000	3000	0.1	0.5	1	5	0.17	0.17	0.05	0.01	0	1

**Table 4:** Result of machine learning model application and comparison with parameters obtained with optimization.

**Parameter**	**Optimal Value**	**Predicted Value**	**Relative Error, %**
** $b,\;V^{-1}$ **	0.76478	0.99832	~30.5
** $a_{1},\;A$ **	2.63644	2.85663	~8.4
** $a_{2},\;A$ **	2.36923	2.94332	~24.2
** $V_{p},\;V$ **	1.49592	0.79846	~46.6
** $V_{n},\;V$ **	1.50254	0.84646	~43.6
** $x_{p}$ **	0.87569	0.73418	~16.2
** $x_{n}$ **	0.45690	0.59444	~30.1

**Table 5:** Optimal parameters of the model obtained by sequential use of the machine learning model and optimization algorithms.

Parameter	Value	Parameter	Value	Parameter	Value	Error Function
** $V_{p},\;V$ **	1.40	$a_{1},\;A$	3.14	$x_{0}$	0.0954	0.101
** $V_{n},\;V$ **	1.57	$a_{2},\;A$	2.79	$x_{1}$	0.25	
** $A_{p}$ **	7357	$b,\;V^{-1}$	0.67	$x_{2}$	1.02	
** $A_{n}$ **	2068	$x_{start}$	$1\times 10^{3}$	$x_{3}$	0.16	
** $x_{p}$ **	0.8	$\sigma_{0}$	0.25			
** $x_{n}$ **	0.17	$\sigma_{1}$	0.61			
** $\alpha_{p}$ **	0.71	$\sigma_{2}$	0.29			
** $\alpha_{n}$ **	11.19	$\sigma_{3}$	1.56			
